# Clinical Application of a Modular Genomics Technique in Systemic Lupus Erythematosus: Progress towards Precision Medicine

**DOI:** 10.1155/2016/7862962

**Published:** 2016-08-30

**Authors:** Eric Zollars, Sean M. Courtney, Bethany J. Wolf, Norm Allaire, Ann Ranger, Gary Hardiman, Michelle Petri

**Affiliations:** ^1^Division of Rheumatology, Medical University of South Carolina, Charleston, SC 29425, USA; ^2^Center for Genomic Medicine, Medical University of South Carolina, Charleston, SC 29425, USA; ^3^Department of Public Health Sciences, Medical University of South Carolina, Charleston, SC 29425, USA; ^4^Biogen Idec, Cambridge, MA 02142, USA; ^5^Division of Rheumatology, Johns Hopkins School of Medicine, Baltimore, MD 21287, USA

## Abstract

Monitoring disease activity in a complex, heterogeneous disease such as lupus is difficult. Both over- and undertreatment lead to damage. Current standard of care serologies are unreliable. Better measures of disease activity are necessary as we move into the era of precision medicine. We show here the use of a data-driven, modular approach to genomic biomarker development within lupus—specifically lupus nephritis.

## 1. Introduction

Systemic lupus erythematosus (SLE) is the prototypical autoimmune disease. Immune tolerance breaks down leading to the immune system attacking normal tissues. Antibodies form that recognize self-antigens and lead to pathologic immune complex deposition. Dysfunction within both the innate and adaptive immune systems leads to increased cytokine production, especially type I interferons, B-cell overproduction of autoantibodies, and T-cell enhancement of these processes. This leads to the host of clinical abnormalities within SLE that includes rashes, oral ulcers, arthritis, inflammation around the heart and lungs, and cytopenias as well as severe renal and neurologic manifestations.

Treatment of the disease requires attention to all of these possible manifestations of disease activity. Any patient, at any time, can develop increased disease activity in any organ. While some manifestations are obvious (thrombocytopenia) others can be more subtle and complicated by other potential etiologies (is the rash or joint pain from lupus or something else?). There are no serologies which are universally useful for all manifestations across all patients. Anti-dsDNA antibodies associate with renal disease activity in a subset of patients but in only a fraction of these patients do changes in the levels predict changes in disease activity [1]. This is true for complement consumption as well. An inflammatory disease is expected to have elevated inflammatory markers (erythrocyte sedimentation rate, ESR, C-reactive protein, and CRP). In SLE, large changes in ESR are informative (with the caveat that infection also raises the ESR) but minor fluctuations are not [2]. Further, many lupus patients maintain a baseline elevated ESR regardless of clinical disease activity. CRP is less often elevated in SLE outside of infection [3]. There is no shortage of attempts at producing better biomarkers for lupus disease activity. Novel autoantibodies (anti-C1q), cell surface markers, and cytokines are all reported [4]. Only complement surface deposition has reached a level of reproducibility to allow inclusion in a commercially available product (AVISE, http://www.exagen.com/).

Exploration of gene expression in SLE began in 2002 with the cytokine specific arrays in Rus et al. [5]. They showed increased expression of inflammatory cytokines and were able to separate lupus patients from healthy controls. A seminal work within this area was by Baechler et al. in 2003 showing the presence of an interferon signature [6]. Further work reproduced the interferon signal but also a curious neutrophil related signature [7]. However, these early works revealed the well-described limitations of microarray work—difficulties with technical and biological variability [8]. Technical variability includes batch effects as well as different probe sequences in different platforms. Biological variability leads to the lack of reproducibility between microarray studies. Furthermore, the interferon signature developed in Baechler was not shown to track disease activity [9].

The limitations in large-scale gene expression analysis lead to the development of gene ontology [10] and functional enrichment methods such as gene set enrichment analysis (GSEA) [11]. These methods involve the annotating of transcripts with known roles in biologic processes and pathways as well as molecular structure and cellular components. These methods were primarily performed within oncologic processes.

Chaussabel and coworkers developed a novel, data-driven method using primarily inflammatory diseases [12]. In this method, clusters of genes that were observed in multiple disease processes were clustered into “modules.” These modules were then labeled based on their primary function based on an automated literature search. This method was shown to discriminate between active and inactive disease within a pediatric lupus cohort. The method, initially developed on Affymetrix arrays was updated for use on Illumina arrays and the module list was greatly expanded. Importantly, the interferon module from 2008 was split into three modules, two of which were responsive to changes in disease activity. Further work by the group, recently published [13], expanded the work further, showing longitudinal variation as well as treatment response. They showed the prominence of a plasma cell signature within a subset of pediatric lupus patients that was a reliable marker of disease activity.

The work here evaluates the utility of the modular approach in an adult lupus population. Banchereau et al. mention that the pediatric population is a special population to study and this is true as patients who present with lupus in the pediatric years are more likely to have severe disease such as lupus nephritis or neurologic disease. Furthermore, we are specifically evaluating how a modular approach can discriminate between active and inactive lupus patients. This is likely a more difficult problem than discriminating between lupus patients and healthy controls. Also, it is unclear whether differences found between lupus and healthy controls can be directly applied to differences within lupus patients.

## 2. Methods 

### 2.1. Study Population and Design

The study protocol for SPARE (Study of biological Pathways, disease Activity and Response markers in patients with systemic lupus Erythematosus) was approved by the Johns Hopkins University School of Medicine Institutional Review Board. SLE patients were enrolled from the Hopkins Lupus Cohort following informed consent. Adult patients were eligible if they were aged 18 to 75 years and met the definition of SLE as defined by the revised American College of Rheumatology classification criteria [14]. At entry into the study the patient's medical history was reviewed and information on current medications was recorded. Visits were scheduled quarterly or more often if required for disease activity over a 2-year period. All patients were evaluated by the same physician at entry and all subsequent cohort visits (MP). Three hundred and six SLE patients were enrolled in the observational study. Patients were treated according to standard clinical practice. To assess disease activity, the Safety of Estrogens in Lupus Erythematosus: National Assessment (SELENA) version of the Systemic Lupus Erythematosus Disease Activity Index (SLEDAI) [15] as well as the Physician Global Assessment [16] was completed at each visit.

### 2.2. Sample Selection

The samples selected for this analysis were chosen retrospectively based on the recorded clinical information. The goal was to compare lupus patients with active disease to lupus patients with clinically quiescent disease. Lupus nephritis is one of the more objective and persistent forms of lupus disease activity. The amount of protein in the urine is a relatively reliable indicator of ongoing inflammation within the kidney (with caveats, [17]). The “high activity” patients were selected based on the presence of a urine protein/creatinine ratio of 1.6 or higher. There was no selection for medications, ethnicity, or age. The “no activity” patients were clinically assessed to have no lupus disease activity (PGA = 0) and were on no immune-modifying medications other than hydroxychloroquine. Standard practice in lupus treatment includes continuing hydroxychloroquine therapy regardless of disease activity. Another comparator group, “typical” lupus, was created to compare specifically to the healthy controls. This group was randomly chosen from the dataset with the only restriction that no patient would be represented more than once. The healthy control patients were collected by Biogen and were never assessed by MP. Patient characteristics are shown in Table 1.

### 2.3. Sample Preparation

Peripheral blood samples used for gene expression analyses were collected using the PAXgene Blood RNA system (PreAnalytix GmbH). RNA was isolated from PAXgene preserved blood using the Agencourt RNAdvance Blood kit automated on an Arrayplex liquid handling system (Beckman Coulter, Indianapolis, IN). RNA integrity and concentration were assessed using the HT RNA reagent kit (Caliper Life Sciences, Hopkinton, MA) using a LabChip GX (PerkinElmer, Waltham, MA). RNA samples with a RQS score of >8.0 were considered of acceptable quality for downstream applications.

### 2.4. Gene Expression Analysis

RNA (50 ng) isolated from the PAXgene blood sample was amplified and biotin-labeled with the NuGEN Ovation RNA Amplification system V2, Ovation WB reagent, and Encore Biotin module (NuGEN Technologies, Inc., San Carlos, CA) using an Arrayplex automated liquid handler (Beckman Coulter, Indianapolis, IN). 2 ug of biotin labeled sscDNA probe was hybridized to Affymetrix GeneChip HT HG-U133+ PM plate arrays with modified conditions as described in Allaire et al. [18]. Washing and staining of the hybridized arrays were completed as described in the GeneChip Expression analysis technical manual for HT plate arrays using the Genechip® Array Station (Affymetrix, Santa Clara, CA) with modifications as described in Allaire et al. [18]. The processed Genechip plate arrays were scanned using GeneTitan scanner (Affymetrix, Santa Clara, CA). Affymetrix scans were subjected to standard quality control (QC) measures. These tests included a visual inspection of the distribution of raw signal intensities and an assessment of RNA degradation, relative log expression (RLE), and normalized unscaled standard error (NUSE). All sample scans passed these QC metrics. CEL files were subjected to GC-content-based Robust Multi-array Average (GCRMA) normalization [19]. Expression levels were log (base 2) transformed. All calculations and analyses were carried out using R and Bioconductor computational tools [20]. Modules used included limma [21], PAMR [22], and GEOQuery [23]. TopGene (https://toppgene.cchmc.org/) was used for functional enrichment analysis. Genes composing Chaussabel modules were taken directly from Table S2 in [12].

## 3. Results

**Figure 1:**
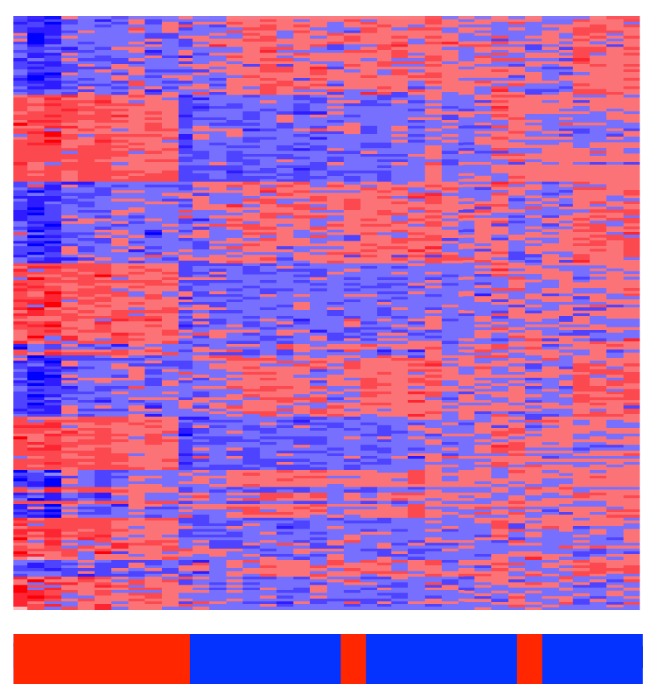
Heatmap of SLE patients. Hierarchical clustering separates patients with high disease activity from those with no clinical disease activity. The red bars along the bottom indicate patients with high disease activity and green bars indicate those with no disease activity.

An analysis of differential gene expression leads to a total of 799 genes differentially expressed at a Benjamini-Hochberg adjusted significance of 0.05. There is an obvious difference between the high and no disease activity groups as seen in the heatmap in Figure 1. For the most part, the high disease activity clusters to the left and the low disease activity clusters to the right. Simple hierarchical clustering separates 10/13 high disease activity from the no disease activity.

Functional enrichment analysis using TopFunn shows results that would be expected for lupus (Table 2). There is significant upregulation of type I interferon pathways, immune pathways, and cytokine-associated pathways. There was nothing unexpected in this analysis.

A search for a “gene signature” that would separate high lupus disease activity from no disease activity used PAMR. This is a clustering method that finds the smallest list of genes that leads to the smallest misclassification error. These genes are shown in Table 2. While there are genes in the list that are biologically plausible, it suffers from “noise” inherent in these microarray gene lists [24].

We next looked at the modules developed by Chaussabel and colleagues. We used the 2008 modules as they were developed using the Affymetrix platform. The arrays used in the study reported here lack the mismatch probes of the U133A and U133B chips used by Chaussabel but are otherwise the same probes. We first reproduced the work of Chaussabel using the datasets available on the NIH GEObus (GSE11909). These were comparing untreated pediatric lupus to healthy controls. This dataset was not complete, missing 6 of 12 U133B chips of the healthy controls, but all lupus and all 133A chips were available. This is shown in Table 3. Each module has a certain number of genes. For instance, Module 1.1, plasma cells, is defined by the membership of 76 genes. The first data box in Table 3 is reproduction of the pediatric lupus data. The number of significantly differentially expressed genes is shown as a proportion. Thus, 45% of the genes within Module 1.1 are increased in expression when comparing untreated pediatric lupus to healthy controls. This nearly exactly reproduces the original work as expected. This work showed the notable increased expression of genes within the interferon, plasma cell, neutrophil, erythrocyte, and myeloid modules. Decreased expression was seen in genes associated with the ribosomal, cytotoxic, and T-cell modules.

We then looked at the modular representation of gene expression differences between high and low disease activity in adult SLE patients. The reason for doing this is that we hypothesized that what distinguishes high and low disease lupus within SLE may be different than what distinguishes SLE from healthy controls. In the second data box, “Adult SLE, High versus No Activity,” we show that 97% of genes within the interferon module are significantly increased in expression. Thus, even within SLE there is increased expression of the genes that make up the interferon module. This is similar to the untreated pediatric lupus population. It is also quite expected as many studies have shown the importance of the interferon pathways in SLE. Remarkably, even in this treated, adult population we see an increase in the neutrophil signature. The neutrophil signature is associated with lupus nephritis [25] and this was also demonstrated in the most recent pediatric SLE study [13]. We do not observe significantly increased numbers of genes associated with the plasmablast signature.

Next we looked at a cohort of adult lupus patients with a typical mixture of disease activity and compared to healthy controls. This is an attempt to make the comparison with Data Box 1 but with adult lupus patients on a mix of therapies. Characteristics of this group are shown in Table 1. The average SLEDAI for this group is 2.7, representing mild disease. Only eight of the 95 patients have renal disease, six have arthritis, and none have significant neurologic disease. Again, we do not see the plasma cell signature reported in Chaussabel et al. [12] and Banchereau et al. [13]. Again, the interferon signal is very strong but notably is not significantly different from either of the other comparisons. There are notable differences between the comparison within lupus patients and the comparison between lupus and healthy controls. For the neutrophil module, 55% of the genes were present in high disease activity while only 29% showed this difference when comparing “typical lupus” to healthy controls. This is roughly what was seen in the pediatric population where there was some evidence that increased expression of genes in the neutrophil module associated with increased disease activity. Increased presence of reduced expression genes was seen in the two ribosomal modules, T-cell module and cytotoxic modules.

## 4. Discussion

The clinical evaluation and treatment of patients with SLE is in desperate need of advanced biomarker development. Assessment of disease activity is difficult and currently inadequate. Medical treatment decisions are, for the most part, not guided by individual characteristics of the patient or the disease. If we are ever to achieve precision medicine in this complex, heterogeneous disease it will be through detailed molecular phenotyping and close monitoring of reliable indicators of disease activity.

Gene expression analysis allows for measurement of many variables at once, potentially allowing for capture of the heterogeneity of this complex disease. Multiple techniques for dimension reduction have been proposed and one of the more promising for the quantification of disease activity in lupus is the modules developed in pediatric lupus by Chaussabel et al. [12]. We show here the application of these modules in quantification of SLE disease activity, specifically lupus nephritis.

The results of this analysis show some similarities with the pediatric lupus patients studied in Dallas [12, 13, 26]. First, the prominence of the interferon signature is reproduced. This is not surprising based on the fundamental importance of interferon in SLE pathology. It is worth noting that a “score” of 97% in the interferon module indicates that 97% of genes in that module were found to have significant differential gene expression. Comparing SLE patients with high disease activity to those with low disease activity reveals increased expression of these genes. However, comparing “typical” SLE activity to healthy controls reveals increased expression as well. The score does not indicate the* level* of gene expression only that there was an increase from one group to another. Microarray measurement of gene expression has some association with more quantitative methods of measuring mRNA, for example qPCR, but is not as sensitive to changes in expression [27]. We did not make an attempt here to further quantify the amount of expression within the module. Further work in this area can include an analysis with RNAseq that leads to actual mRNA counts.

Another similarity with the pediatric study is the presence of the neutrophil module. Truly remarkable work evaluating the roles of neutrophils in SLE pathology emerged after the demonstration of the neutrophil signature [7]. Unlike the interferon module above, the neutrophil module did show increased numbers of differentially expressed genes in the active-inactive group compared to the lupus-healthy control group. This is possibly due to the demonstrated role of neutrophils in lupus nephritis specifically [25]. However, it is also possible that multiple neutrophil-related interactions are involved. Perhaps increased disease leads to recruitment of more of these pathways or that some patients have different components of these pathways.

A significant difference between this work and the original work of Chaussabel is in the plasma cell module. Plasma cells are the antibody producing cells and are increased in active lupus. In the recent work with the Dallas pediatric SLE cohort the plasmablast module was found to associate with disease activity and was significantly reduced by treatment with mycophenolate [13]. We find here, in an adult population, the absence of a significant plasma module differentiating either active from inactive disease or typical SLE from healthy controls. This seems to be also true in the primarily Caucasian, French population analyzed in Chiche et al. [26]. In that work, the newer Illumina-based modules were used where the plasma cell module is Module 4.11. Thus, it is unclear at this point if the plasma cell signature is enhanced in the pediatric population studied or unable to be differentiated from treatment effects in this study.

This study has multiple limitations. First, for a true biomarker study the response to change in disease activity and outcomes would have to be followed over time. At this time we were interested in the applicability of the modules in a specific subpopulation of lupus but plan for further analysis. The limited size of this sample did not allow for treatment effects to be studied, though great effort was made to minimize those differences in the active versus inactive group. Finally, one of the limitations is that the technology of the Affymetrix microarray is limiting and future work will include more quantitative and reproducible techniques.

There is great promise in the use of data-driven analysis in the exploration of complex, heterogeneous diseases such as lupus. We show here an example of how this can be used in evaluating active versus inactive disease within SLE. As we move to precision medicine methods such as these will lead to better characterization of disease, better therapies, and better response to therapies.

## Figures and Tables

**Table 1:** Patient characteristics.

	High activity *N* = 13	Low activity *N* = 25	Healthy *N* = 51	Typical SLE *N* = 95
Average age (SD)	43.9 (12)	44 (14)	39 (11)	46 (12)
Ethnicity (%)				
African American	23	44	27	38
White	62	52	73	58
Average SLEDAI	7	0	NA	2.7
Mycophenolate (N)	6	0	NA	21
Azathioprine (N)	2	0	NA	12
Average Prednisone (mg)	8.5	0	NA	3

**Table 2:** Functional enrichment analysis from ToppGene. Hit count in query list is the number of genes from the list of significantly differentially expressed genes in that ontology. Hit count in genome is the number of described genes in the ontology.

ID	Name	*p* value	*q*-value Bonferroni	Hit count in query list	Hit count in genome
GO:0006955	*Immune response*	1.35*E* − 25	6.46*E* − 22	114	1416
GO:0019058	Viral life cycle	9.43*E* − 24	4.50*E* − 20	50	314
GO:0045087	Innate immune response	2.20*E* − 23	1.05*E* − 19	84	883
GO:0044764	Multiorganism cellular process	4.44*E* − 23	2.12*E* − 19	75	725
GO:0071357	*Cellular response to type I interferon*	4.70*E* − 23	2.25*E* − 19	27	76
GO:0060337	*Type I interferon signaling pathway*	4.70*E* − 23	2.25*E* − 19	27	76
GO:0051607	Defense response to virus	6.08*E* − 23	2.90*E* − 19	41	212
GO:0034340	*Response to type I interferon*	7.06*E* − 23	3.37*E* − 19	27	77
GO:0016032	Viral process	3.62*E* − 22	1.73*E* − 18	73	714
GO:0009615	Response to virus	1.85*E* − 21	8.84*E* − 18	47	310
GO:0002252	Immune effector process	2.18*E* − 21	1.04*E* − 17	67	628
GO:0006952	Defense response	1.45*E* − 19	6.93*E* − 16	107	1515
GO:0098542	Defense response to other organisms	5.35*E* − 19	2.56*E* − 15	50	401
GO:0043207	Response to external biotic stimulus	1.29*E* − 18	6.14*E* − 15	68	726
GO:0051707	Response to other organisms	1.29*E* − 18	6.14*E* − 15	68	726
GO:0009607	Response to biotic stimulus	1.43*E* − 17	6.82*E* − 14	68	760
GO:0048525	Negative regulation of viral process	1.43*E* − 16	6.85*E* − 13	22	78
GO:0034097	*Response to cytokine*	2.85*E* − 16	1.36*E* − 12	59	629
GO:0071345	*Cellular response to cytokine stimulus*	5.70*E* − 16	2.72*E* − 12	53	527
GO:0006414	Translational elongation	2.32*E* − 15	1.11*E* − 11	26	130
GO:0045069	Regulation of viral genome replication	1.31*E* − 14	6.27*E* − 11	18	58
GO:0006413	Translational initiation	1.87*E* − 14	8.94*E* − 11	29	179
GO:0019221	*Cytokine-mediated signaling pathway*	4.42*E* − 14	2.11*E* − 10	43	402
GO:0019079	Viral genome replication	1.94*E* − 13	9.25*E* − 10	19	76
GO:0043900	Regulation of multiorganism process	4.19*E* − 13	2.00*E* − 09	37	325
GO:0035455	*Response to interferon-alpha*	4.56*E* − 13	2.18*E* − 09	11	19

**Table 3:** Module name and number as described in [12]. The “pediatric SLE” column is reproduced from GSE11909. The numbers shown are the percentage of genes from the module that have significant differential gene expression (DGE). Thus, the first row is interpreted as follows: 45% of the genes in Module 1.1 have significant DGE and are increased in expression. The second column, “Adult SLE, High versus No Activity,” is a DGE analysis between lupus patients with high disease activity and lupus patients with no activity. The third column, “Adult SLE versus HC” is a DGE analysis between a cohort of lupus patients with average disease activity and healthy controls.

Module name	Module number	Pediatric SLE Untreated versus HC		Adult SLE High versus no activity		Adult SLE SLE versus HC	
		Up	Down	Up	Down	Up	Down
Plasma cells	1.1	45	0	8	0	4	14
Erythrocytes	1.2	5	5	5	9	20	7
B-cells	1.3	1	4	3	36	1	30
None	1.4	5	4	5	18	8	19
Myeloid	1.5	13	6	9	15	32	4
None	1.6	5	5	1	11	0	45
Ribosomal	1.7	0	78	5	64	11	40
None	1.8	3	5	2	27	6	21
Cytotoxic	2.1	2	21	1	40	2	29
Neutrophils	2.2	33	0	55	0	29	4
Erythrocytes	2.3	26	3	9	3	9	8
Ribosomal	2.4	0	77	0	74	5	36
None	2.5	1	27	3	3	6	5
Myeloid	2.6	23	2	10	3	28	8
None	2.7	1	31	0	4	1	7
T-cells	2.8	0	42	0	56	1	32
None	2.9	3	12	8	1	3	40
None	2.1	4	8	8	11	18	8
None	2.11	2	6	2	8	4	22
Interferon	3.1	91	0	97	0	97	0
Inflammation	3.2	13	2	16	4	20	14
Inflammation	3.3	9	6	8	7	17	8
None	3.4	6	14	2	16	2	25
None	3.5	14	18	5	5	14	5
None	3.6	6	5	2	15	2	19
None	3.7	4	9	1	23	17	9
None	3.8	1	13	1	45	4	42
None	3.9	2	11	1	29	3	23
